# Regulatory T Cells and Profile of FOXP3 Isoforms Expression in Peripheral Blood of Patients with Myelodysplastic Syndromes

**DOI:** 10.1155/2018/8487403

**Published:** 2018-10-10

**Authors:** Galina A. Dudina, Almira D. Donetskova, Marina M. Litvina, Alexander N. Mitin, Tatiana A. Mitina, Sergey A. Polyakov

**Affiliations:** ^1^Loginov Moscow Clinical Center of the Moscow Health Department, 111123 Moscow, Russia; ^2^National Research Center–Institute of Immunology Federal Medical-Biological Agency of Russia, 115522 Moscow, Russia; ^3^Pirogov Russian National Research Medical University, 117997 Moscow, Russia; ^4^Moscow Regional Research Clinical Institute Named after MF Vladimirsky, 129110 Moscow, Russia; ^5^Celgene International Holdings Corporation, 125047 Moscow, Russia

## Abstract

We have investigated the frequencies of regulatory T cells and the level of FOXP3 isoforms expression in peripheral blood of patients with myelodysplastic syndromes and found the significant reduction of regulatory T cells at all stages of the disease. At the same time in untreated patients, we observed the shift in the FOXP3 isoforms expression profile towards the full-length molecule possibly due to inflammation. Based on the already known information about the potentially higher functional activity of FOXP3 molecule lacking exon 2, we have also hypothesized that our finding may explain the high risk of autoimmune disorders in this disease.

## 1. Introduction

Myelodysplastic syndrome (MDS) is a heterogeneous group of diseases, caused by clonal stem cell disorders, with the specific sign of peripheral cytopenia due to ineffective hemopoiesis with normal or increased cellularity of the bone marrow. The clinical manifestations, course, and outcome of MDS are highly diverse, and the median survival varies from 6 months to 5 years [1]. MDS has always been viewed through the prism of clonal expansion of hematopoietic progenitor cell with further risk of transformation into acute myeloid leukemia (AML) in approximately 30–40% of patients [2]. Despite the existence of prediction scales with a well-defined prognostic structure based on cytological and cytogenetic laboratory parameters the course of the disease and the progression of leukemic infiltration are often very unpredictable. The problems of predicting acute leukemia, in turn, make it difficult to select the treatment tactics. The number of clinical studies aimed at investigating new approaches to stratifying the risk of the disease progression is growing every year.

Considering that the immune system plays an active role in the pathogenesis of MDS, one can assume that some immunological parameters, for example, the number of regulatory T cells (Treg), can be used as prognostic criteria. To some extent participation of Treg in MDS pathogenesis can explain the association of this disease with both autoimmune disorders [3] and tumor transformation [1], considering that low quantity and decreased function of Treg lead to weak suppression of excessive immune response, while a high number and increased function of Treg can lead to disruption of the immune surveillance of tumor growth.

Most of the conducted studies link increased Treg frequencies with an unfavorable MDS prognosis [4–7]. Despite a similar conclusion in these studies, the data obtained on the number of Treg in MDS were rather contradictory, probably relating to different sample preparation protocols and gating strategies used in flow cytometry analysis [8]. This assumption is indirectly confirmed by differences in Treg frequencies in the age-matched healthy donors.

Attempts to use functional Treg characteristics as a prognostic criterion for MDS have also been made. Mailloux et al. have demonstrated that an increased number of Treg with the effector memory T-cells phenotype correlated with a poor prognosis of MDS, such as transformation into acute myeloid leukemia and low survival [9]. However, the obtained results may not be so much a prognostic criterion but the reflection of a specific stage of the disease.

Before considering the functional Treg characteristics as a prognostic criterion for MDS, one must take into account that the main regulator of Treg differentiation and function is the FOXP3 transcription factor [10]. So the features of its expression should have a significant effect on the Treg function. In the studies of the molecular structure of FOXP3, it has been determined that alternative splicing in humans results in four mRNA variants and four isoforms of FOXP3: the full-length molecule (FOXP3-FL); with exon 2 deletion (FOXP3Δ2); with exon 7 deletion (FOXP3Δ7); and with simultaneous deletion of exons 2 and 7 (FOXP3Δ2Δ7) [11–13]. In a recently published review [14], Mailer R. analyzes in detail the biology of FOXP3 alternative splicing and the specific functions of FOXP3 isoforms. Functional significance of the regions encoded by the deleted exons is different. In brief, exon 2 encodes the FOXP3 domain responsible for binding transcription factors of ROR*α* and ROR*γ*t families [15, 16] that determine the proinflammatory Th17 polarization of the immune response; exon 7 encodes the sequence responsible for FOXP3 dimerization, and its absence disrupts the Treg suppressor function [13, 17]. An essential feature of FOXP3 molecules lacking exon 2 and 7 products is their preferential localization within the nucleus: Magg et al. have shown that they lose nuclear export signal (NES) sequences located in the regions encoded by exons 1/2 and 6/7 [18]. This group has also demonstrated that FOXP3 expression is mainly detected in the cytoplasm upon activation of naive CD4^+^CD25^−^ T cells, in contrast to a predominant localization in the nucleus in CD4^+^CD25^+^ Treg [18]. Localization of FOXP3 within the nucleus is very crucial for its function as a transcriptional activator and suppressor. Therefore, we can assume that FOXP3Δ2, which has a suppressor function and is located predominantly in the nucleus, is the dominant isoform that determines Treg functional activity.

Considering the inconsistency of the available data on the number of Treg, their potentially important role in MDS pathogenesis, and functional differences between expressed FOXP3 isoforms, we decided to evaluate not only the number and percentage of Treg in this disease but also the level of FOXP3 isoforms expression in patients with MDS at different stages of the disease.

## 2. Materials and Methods

Seventy-six MDS patients were enrolled in the study (Table 1). They were divided into three groups: primary (MDS-primary), early-stage (E-MDS), and late-stage MDS (L-MDS). Patients before treatment (any stage of disease) represented the MDS-primary group. E-MDS and L-MDS groups consisted of pretreated patients. They were divided according to the International Prognostic Scoring System (IPSS). Using blast percentage, karyotype, and number of cytopenias, this scoring system reliably estimates survival and risk of leukemic transformation [1]. In the IPSS, cytopenias were defined as hemoglobin < 10 g/dL, absolute neutrophil count < 1.83 x10^9^/L, and platelet count < 100x10^9^/L. Cytogenetic categories were as follows: good (normal, -Y, del (20q), del (5q)), poor (chromosome 7 abnormalities, and complex which is defined as 3 or more abnormalities), and intermediate (all other abnormalities). Pretreated patients who scored less than or equal to 1.0 according to IPSS (low and intermediate-1 risk groups) were assembled into E-MDS group. Pretreated patients who scored 1.5 and more according to IPSS (intermediate-2 and high-risk groups) were assembled into L-MDS group. MDS-primary group included 21 patients (14 women, 7 men) and age median was 72.0 (64–76.5) years. E-MDS group included 27 patients (15 women, 12 men), age median was 71.5 (64–76) years. L-MDS group included 28 patients (12 women, 16 men), age median was 68.5 (63–73) years. Twenty-six age-matched healthy donors (15 women, 11 men), age median of 72 (48–79) years, were enrolled as an age control group (Table 1). Thus, the patient characteristics were similar to each other in the age and sex and corresponded to the age control group. All patients from the E-MDS and L-MDS groups were red blood cell (RBC) transfusion dependent. The blood transfusion burden ranged from 2–3 to 5–6 packed RBC units per month. Patients had been receiving RBC transfusions from 4 months to 5 years. The study was conducted before prescription of hypomethylating or cytostatic therapy even in the L-MDS group.

MDS patients (up to 20% blasts) of any IPSS risk aged 18 years and more who signed informed consent form were included in the study. Patients with other malignancies, with severe uncontrolled cooccurring chronic and recurrent diseases, pregnant or breastfeeding women, and patients with psychiatric disorders making the patient unable to sign informed consent were excluded.

The study material was peripheral blood. Blood samples were collected into the test tubes with an anticoagulant. Cells were counted on a hematology analyzer according to the conventional technique. Isolation of peripheral blood mononuclear cells (PBMCs) and their subsequent analysis were carried out within the next 6 hours.

PBMCs were isolated by centrifugation in a Ficoll density gradient (1.077 g/cm^3^), suspended in PBS with 1% BSA and 0.01% NaN_3_ (washing and incubation buffer), and incubated with monoclonal antibodies (MAbs) against surface markers for 30 min at 4°C. Then cells were washed and permeabilized for 40 min in Foxp3 Fixation/Permeabilization Buffer (eBioscience) according to the company's methodological guidelines, washed again and incubated for 90 min at 4°C in the dark with anti-FOXP3 MAbs, washed for the last time, and analyzed immediately using a flow cytometer. As Treg is a minor population, at least 2×10^5^ cells entering the lymphocyte gate were analyzed, and this made it possible to reduce the error. Flow cytometry was performed on a BD FACSCanto™ II flow cytometer (Becton Dickinson) in the standard mode. The data were analyzed using FlowJo software (Treestar).

**Figure 1:**
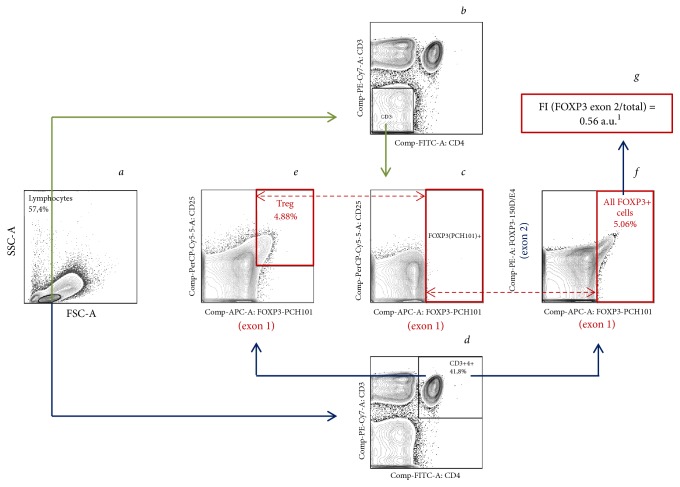
Flow cytometry. The algorithm of regulatory T cells gating using PBMCs^2^ of the 74-year-old healthy donor. (*a*) Lymphocytes among PBMCs. (*b–c*) Establishing FOXP3^+^ gate using CD3^−^ cells known not to express FOXP3: CD3^−^ cells among lymphocytes (*b*); establishing FOXP3 (exon 1)^+^gate using CD3^−^ cells (*c*). (*d–e*) Detection of Treg: CD4^+^ T cells among lymphocytes (*d*); CD25^+^ FOXP3 (exon 1)^+^ – Treg among CD4^+^ T cells (*e*). (*f–g*) Analysis of FOXP3 isoform expression: all FOXP3^+^ cells among CD4^+^ T cells (*f*); calculation of FI (FOXP3 exon 2/total) parameter in all FOXP3^+^ cells = [(FOXP3 exon 2 MFI)/(FOXP3 exon 1 MFI)] (*g*). The solid arrows show the gating algorithm. The dashed bidirectional arrows show the alignment of the established gates for CD3^−^ and CD3^+^CD4^+^ cells. ^1^a.u.: arbitrary units; ^2^PBMCs: peripheral blood mononuclear cells.

MAbs labeled with different fluorophores,* i.e.*, FITC (fluorescein isothiocyanate), PE (phycoerythrin), APC (allophycocyanin), PerCP-eFluor 710 (peridinin-chlorophyll-protein-eFluor 710), and PE-Cy7 (phycoerythrin-cyanin7), were used. The following combination of MAbs manufactured by eBioscience was used (isotype controls from the same company): CD3-PE-Cy7, CD4-FITC, CD25-PerCP-eFluor710, FOXP3 (PCH101)-APC, and FOXP3 (150D/E4)-PE. The peculiarity of this technique is the epitope specificity of anti-FOXP3 MAbs. The PCH101 MAbs recognize the FOXP3 epitope encoded by exon 1; in other words, all FOXP3 isoforms, and the 150D/E4 MAbs recognize the epitope encoded by exon 2, i.e., FOXP3-FL and FOXP3Δ7 exclusively. The gating algorithm for discrimination of Treg is presented in Figure 1,* a*,* d, e,* and all FOXP3^+^ cells are presented in Figure 1,* a*,* d, f*. Previous studies have shown that it is impossible to divide the FOXP3^+^ population according to the level of FOXP3 isoforms expression and it is useful to estimate the ratio of the appropriate fluorescent intensities in the total FOXP3^+^ population [19, 20]. Thus we determined the mean fluorescent intensities (MFI) of fluorophore-conjugated MAbs binding to FOXP3 exon 2 (FOXP3 exon 2 MFI) and exon 1 (FOXP3 exon 1 MFI) in all CD3^+^CD4^+^FOXP3^+^ cells to derive parameter FI (FOXP3 exon 2/total) = [(FOXP3 exon 2 MFI)/(FOXP3 exon 1 MFI)] (Figure 1, f, g), as it was described previously [19]. CD3^−^ cells that do not express FOXP3 were used as a negative control for the establishment of the gate for FOXP3^+^ cells (see Figure 1,* a–c*).

The statistical analysis was performed using nonparametric statistics. The parameters were presented as* Me *(*L-H*), where* Me* is the median,* L* is the lower quartile, and *H* is the upper quartile. We used Kruskal-Wallis test to analyze four groups. In case of statistically significant differences between groups, we used Mann–Whitney *U* test to compare the quantitative characteristics of the two groups. The relative frequencies were presented using 95% confidence intervals as X [X1; X2], where X was a frequency and X1 and X2 were the lower and upper confidence limits, respectively. We used two-tailed Fisher's exact test for comparing two independent binomial proportions. A p value less than 0.05 was considered statistically significant. Data analysis was performed with StatSoft Statistica v.12.0.

## 3. Results

Seventy-six patients with the verified diagnosis of MDS were examined in accordance with the World Health Organization classification for tumors of the hematopoietic and lymphoid tissues (2008) (Table 2). 14 patients had refractory anemia (RA), six had an isolated deletion of the long arm of chromosome 5 (del(5q)), 5 had refractory anemia with ring sideroblasts (RARS), 17 had refractory cytopenia with multilineage dysplasia (RCMD), 15 had refractory anemia with excess blasts 1 (RAEB-1), and 19 had RAEB-2. 48 patients had no karyotype changes, and 28 had the following chromosomal abnormalities: 6 patients, del(5q); 4 patients, del(7q); 2 patients, del(20q); 2 patients, del(Y), 8 had abnormalities of two chromosomes, and 6 had more than three clonal chromosome rearrangements. In accordance with IPSS all MDS population was classified into low-risk (19 patients), intermediate-1 risk (12 patients), intermediate-2 risk (19 patients), and high-risk groups (26 patients). Pretreated patients divided by this criterion in the two groups (E-MDS and L-MDS) were represented as follows: low-risk, 13 patients; intermediate-1 risk, patients 14; intermediate-2 risk, patients 10; and high-risk groups, 18 patients. It should be noted beforehand that all the results obtained for the E-MDS and L-MDS groups had no statistically significant differences, so in the text below the significance of the differences is mentioned only in the context of a comparison with the MDS-primary or age control groups. However, we did not join E-MDS and L-MDS groups to emphasize the absence of these differences in our study, despite previous findings linking a poor prognosis of the disease with increased Treg frequencies [4–7].

**Figure 2:**
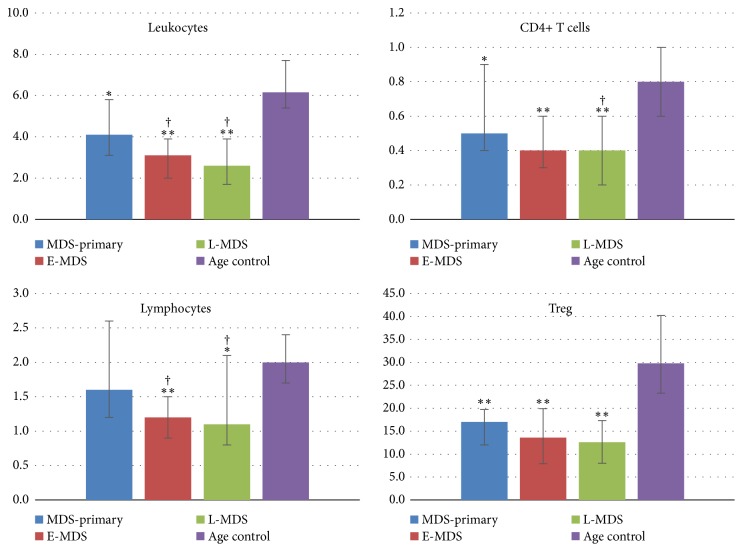
The absolute number of cell populations in the peripheral blood of MDS patients and age-matched healthy donors (10^9^ cells/L, and Treg: 10^6^ cells/L). Note: *∗p*<0.05 rel. to age control; *∗∗p*<0.001 rel. to age control; ^†^p<0.05 rel. to MDS-primary. MDS-primary: primary myelodysplastic syndrome; E-MDS: early-stage myelodysplastic syndrome; L-MDS: late-stage myelodysplastic syndrome groups.

In all MDS groups, the absolute number of leukocytes, lymphocytes, and CD4^+^ T cells in peripheral blood was reduced in comparison with the age control group (Table 3), which is typical for MDS. In pretreated patients (E-MDS and L-MDS groups), the decrease in the number of leukocytes and lymphocytes became more notable in comparison with the primary patients. In addition, there was a significant decrease in the number of CD4^+^ T cells in the L-MDS group. However, in contrast to the previously published data on the possible increase in the number of Treg in certain cases of MDS [3–6], we observed an approximately twofold decrease in the absolute number of Treg in all MDS groups compared to the age control group (Table 4). It is necessary to clarify that only cells with CD3^+^CD4^+^CD25^+^FOXP3^+^ phenotype were considered as Treg. FOXP3 expression, in this case, was determined by the binding of cells with PCH101 MAbs (Figure 1,* e)* which detect all FOXP3 molecules. The quantitative decrease of Treg was proportional to the degree of leukopenia and somewhat more notable than the reduction in the number of lymphocytes and all CD4^+^ T cells, which is evident in the graphs with the absolute number of cell populations in the peripheral blood of MDS patients (Figure 2). The more notable Treg reduction in comparison with all CD4^+^ T cells was due to a decrease in the percentage of Treg among CD4^+^ T cells (Table 4). In MDS-primary and E-MDS groups, the Treg percentage reduction was statistically significant, suggesting a higher probability of developing autoimmune disorders in these MDS groups. In general, the decrease in the number of Treg, regardless of the stage of disease, reflects an overall decline in the number of cells of bone marrow origin in the periphery and is a sign of disrupted hemopoiesis in MDS.

**Figure 3:**
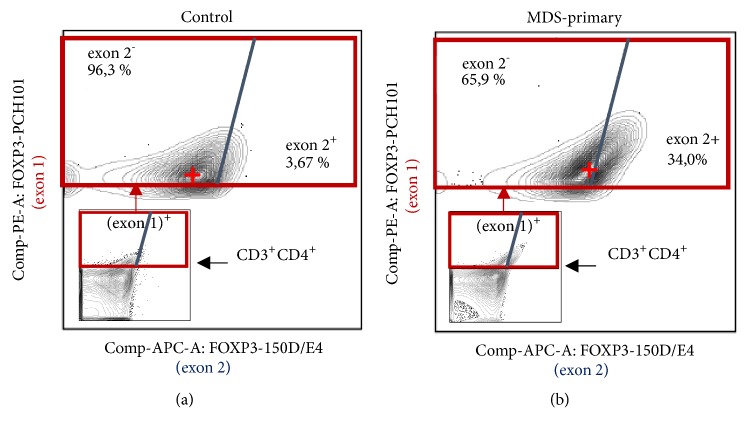
Flow cytometry. Contour plots reflecting FOXP3 exon 1 and exon 2 coexpression in CD3^+^CD4^+^ cells (small plots) and CD3+4+FOXP3+ cells (large plots) from PBMC^1^ of healthy age-matched donor (a) and primary patient with MDS (b). The red cross marks the medians of the corresponding fluorescent intensities in the total FOXP3^+^ gate. ^1^PBMCs: peripheral blood mononuclear cells.

Assuming that disturbance of lymphopoiesis as part of hemopoiesis affects not only the quantity but also the function of the cells, we investigated the level of FOXP3 isoforms expression in Treg, which has different functional properties, as we noted above. To this end, we analyzed the MFI of FOXP3 exon 2 and FOXP3 exon 1 in all CD3^+^CD4^+^FOXP3^+^ cells and calculated the parameter FI (FOXP3 exon 2/total) as have been described in Materials and Methods. We have found that MFI of FOXP3 exon 2 in the MDS-primary group increased in comparison with the age control group. This difference of exon 2 fluorescent intensities between primary MDS patient and healthy age-matched donor is clearly visible on the flow cytometry plots reflecting exon 1 and exon 2 coexpression in CD3^+^CD4^+^ cells (Figure 3). After calculation of FI (FOXP3 exon 2/total) parameter (Table 5) we have found that the ratio of FOXP3-FL to all FOXP3 was significantly increased in the MDS-primary group relative to the age control group, but after treatment, in E-MDS and L-MDS groups, the ratio returned to normal. Thus, the diminished frequency of Treg in MDS is accompanied by the relative accumulation of FOXP3-FL, and this FOXP3 isoforms imbalance disappears after the treatment, although the amount of Treg remains unchanged.

## 4. Discussion

Our study has revealed the decrease in frequency and likely functional activity of Treg in MDS. We suppose that the reduction of Treg functional activity in MDS patients is associated with changing the ratio of FOXP3 isoforms expression in favor of FOXP3-FL. The likely reason for the relative accumulation of FOXP3-FL is the inability of Treg to suppress excessive immune response and inflammation that develop in primary MDS patients. Our speculation is based on the following. First, Wang et al. have shown that FOXP3 can be transiently expressed in stimulated nonregulatory CD4^+^ T cells [21]. Second, Lundberg et al. have shown that T cell receptor stimulation induced FOXP3-FL expression in CD4^+^ T cells* in vitro* [19]. Additionally, they have determined that the coronary artery disease, one of the chronic inflammatory diseases, is associated with the aforementioned FOXP3 isoforms expression pattern defined in PBMCs of the patients with this diagnosis [19]. Third, in our study, the relative increase of FOXP3-FL in primary patients with MDS was abrogated by treatment. In other words, this increase was transient.

Another explanation for the possible decrease in the functional activity of Treg cannot be ruled out. It is based on the already known information about the functional properties of different FOXP3 isoforms [13–18]. Assuming that FOXP3-FL predominant pattern is intrinsic for Treg in MDS and its correction in treated patients is associated with the direct effect of treatment on Treg, we can hypothesize that it is the altered FOXP3 isoforms expression that determines the functional activity of Treg.

Indirectly, the relationship of Treg functional activity and FOXP3 isoforms expression is confirmed by data on the level of FOXP3 isoforms expression in Treg during their differentiation in the human thymus [22], as well as in multiple myeloma [23] and chronic inflammatory bowel diseases [20]. A similar technique using MAbs specific to the exon 1 and the exon 2 was employed in those studies to determine the FOXP3 isoforms expression. Here we need to make a significant remark. In our previous studies, we classified Treg subpopulations as FOXP3 exon 2^+^ and exon 2^−^ [22, 23] as some other investigators did [24–26]. Now we consider that it was incorrect. In this discussion, we will try to reinterpret those data. With the formal setting of the gates for the FOXP3 exon 2^+^ and exon 2^−^ cells, as shown in Figure 3, increase in the percentage of the FOXP3 exon 2^+^ cells is accompanied by the increase in the corresponding MFI predominantly in the channel reflecting the FOXP3-FL expression. This fact allows us to interpret the rise in the percentage as the relative accumulation of FOXP3-FL in the FOXP3^+^ cells.

According to the published data, during differentiation in the thymus, the percentage of Treg precursors expressing FOXP3-FL declined as they matured from 65% in the double-positive CD25^+^ thymocytes to 33% in the single-positive CD4^+^ Treg [22]. A new interpretation allows us to think that FOXP3-FL dominates in CD4^+^CD8^+^CD25^+^FOXP3^+^ thymocytes and FOXP3Δ2 predominates in CD3^+^CD4^+^CD25^+^FOXP3^+^ thymocytes. Thereby the functional maturation of Treg in the human thymus is accompanied by the accumulation of FOXP3Δ2.

The further accumulation of FOXP3Δ2 most likely accompanies oncological diseases. For instance, previously we have demonstrated a twofold increase in the number of Treg in the peripheral blood of primary patients with multiple myeloma [23]. Accumulation of FOXP3Δ2 in Treg, maintained even in the remission after Treg quantity normalization, accompanied this increase [23]. These data indicate that FOXP3Δ2 is involved in the pathogenesis of multiple myeloma and, possibly, has a higher functional activity than FOXP3-FL.

Another indirect confirmation of the high functional activity of the FOXP3Δ2 was obtained in the study of chronic inflammatory bowel diseases (IBD) [20]. Initially, the authors have assumed that Th17 polarization of the immune response in IBD may be caused by the accumulation of Treg expressing exclusively FOXP3Δ2 because of an inability of FOXP3Δ2 to bind ROR*γ*t. Nevertheless, no difference in the expression pattern of FOXP3Δ2 relative to FOXP3-FL was seen in the* lamina propria* of patients with Crohn's disease and nonspecific ulcerative colitis versus non-IBD controls. Against a background of a general increase in the quantity of Treg and Th17 polarization in IBD, the absence of FOXP3Δ2 accumulation indicates that the accompanying inflammation is not associated with this isoform. Thus, the opportunity of FOXP3Δ2 to accumulate in the nucleus is more significant when suppressing the immune response than the inability to restrict IL-17 expression.

At the end of the discussion, it is necessary to clarify the discrepancy between our and the earlier obtained data on the Treg amount in MDS. It is possible that, in addition to the differences in sample preparation protocols and flow cytometry strategy, we evaluated the results differently. We did not attempt to link individual fluctuations in the Treg population that were also present in our study with the disease prognosis but used the already available IPSS evaluation system to divide the patients into groups. Therefore, we have demonstrated a trend common for all groups, including the primary patients, towards a decrease in the number and possibly functional impairment of Treg. The results obtained earlier undoubtedly indicate a poor disease prognosis with an increase in the Treg number but are more likely to be a particular case rather than a general trend. These differences can be explained by heterogeneity of diseases united under the common name of MDS that are often manifested by secondary immunodeficiency due to impaired hemopoiesis in the altered bone marrow niche. The nature of the developing dysplasia, which leads to disruption of the well-defined interactions between cells of bone marrow origin, especially those related to the immune system, determines the pathogenesis and clinical symptoms of the disease. The vector of these disorders is determined by what the differentiation stage was initially affected. It is possible that a detailed study of the mechanisms underlying the MDS pathogenesis will lead to an isolation of individual diseases with characteristic clinical features and outcome.

## 5. Conclusion

We have shown the decrease in the absolute number and the percentage of Treg among CD4^+^ T cells in the peripheral blood of all patients with MDS. In untreated patients, the Treg number reduction was accompanied by the relative accumulation of FOXP3-FL that could reflect the presence of inflammation and a decrease in the functional activity of Treg. These observations could explain the high risk of autoimmune disorders in this disease and would be useful for further understanding the role of Treg and FOXP3 isoforms in the pathogenesis of MDS.

## Figures and Tables

**Table 1:** Groups of patients with MDS.

Group	MDS-primary,	E-MDS,	L-MDS,	Age control,
	*n*=21	*n*=27	*n*=28	*n*=26
Age, years	72 (64–76.5)	71.5 (64-76)	68.5 (63-73)	72 (48-79)
*p*	0.53	0.67	0.96	-

Sex				
Male	7 (33.3%)	12 (44.4%)	16 (57.1%)	11 (42.3%)
Female	14 (66.7%)	15 (55.6%)	12 (42.9%)	15 (57.7%)
*p*	0.56	1.0	0.41	-

Note: *p* value is pointed relatively to age control group (two-tailed Fisher's exact test for sex, Mann-Whitney U test for age).

**Table 2:** The characteristics of patients with MDS.

Parameter	Patients with MDS, *n*=76	Frequency, %
Age, years	69 (63–76)	-
Sex		
Male	35	46.1 [34.8; 57.3]
Female	41	53.9 [42.7; 65.2]
MDS variant (WHO2008)		
RA^1^	14	18.4 [9.7; 27.1]
MDS associated with isolated	6	7.9 [1.8; 14.0]
del(5q)	5	6.6 [1.0; 12.2]
RARS^2^	17	22.4 [13.0; 31.7]
RCMD^3^	15	19.7 [10.8; 28.7]
RAEB^4^-1	19	25.0 [15.3; 34.7]
RAEB-2		
Karyotype		
Normal	48	63.2 [52.3; 74.0]
Abnormal*∗*	28	36.8 [26.0; 47.7]
IPSS		
Low	19	25.0 [15.3; 34.7]
Intermediate-1	12	15.8 [7.6; 24.0]
Intermediate-2	19	25.0 [15.3; 34.7]
High	26	34.2 [23.5; 44.9]

Note: *∗*The karyotype changes are described in the text. ^1^RA, refractory anemia; RARS, refractory anemia with ring sideroblasts; RCMD, refractory cytopenia with multilineage dysplasia; RAEB, refractory anemia with excess blasts.

**Table 3:** The absolute number of cell populations in the peripheral blood of patients with MDS and age-matched healthy donors (10^9^ cells/L).

Group	Leukocytes	Lymphocytes	CD4^+^ Т cells
Age control, n=26	6.15 (5.4–7.7)	2.0 (1.7–2.4)	0.8 (0.6–1.0)
MDS-primary^1^, n=21	4.1*∗* (3.0–5.8)	1.6 (1.2–2.6)	0.5*∗* (0.4–0.9)
E-MDS^2^, n=27	3.1*∗∗*^†^ (2.0–3.9)	1.2*∗∗*^†^ (0.9–1.5)	0.4*∗∗* (0.3–0.6)
L-MDS^3^, n=28	2.6*∗∗*^†^ (1.7–3.9)	1.1*∗*^†^ (0.8–2.1)	0.4*∗∗*^†^ (0.2–0.6)

Note: *∗p*<0.05 rel. to age control; *∗∗p*<0.001 rel. to age control; ^†^p<0.05 rel. to MDS-primary. ^1^MDS-primary, primary myelodysplastic syndrome; ^2^E-MDS, early-stage myelodysplastic syndrome; ^3^L-MDS, late-stage myelodysplastic syndrome.

**Table 4:** The absolute number and percentage of regulatory T cells in the peripheral blood of patients with MDS and age-matched healthy donors.

Group	Treg, 10^6^ cells/L	Percentage of Treg among CD4^+^ T cells, %
Age control, n=26	29.8 (23.3–40.2)	4.1 (3.4–4.3)
MDS-primary^1^, n=21	17.0*∗∗* (12.0–19.7)	3.2*∗* (2.7–4.0)
E-MDS^2^, n=27	13.6*∗∗* (7.9–19.9)	3.1*∗* (1.8–4.7)
L-MDS^3^, n=28	12.6*∗∗* (8.0–17.3)	3.6 (2.8–5.8)

Note: *∗p*<0.05 rel. to age control; *∗∗p*<0.001 rel. to age control. ^1^MDS-primary, primary myelodysplastic syndrome; ^2^E-MDS, early-stage myelodysplastic syndrome; ^3^L-MDS, late-stage myelodysplastic syndrome.

**Table 5:** The ratio of FOXP3 exon 2 fluorescence intensity to FOXP3 total fluorescence intensity in CD3^+^CD4^+^FOXP3^+^ cells from the peripheral blood of the patients with MDS and age-matched healthy donors.

Group	FI (FOXP3 exon 2/total), a.u.^4^
Age control, n=26	0.52 (0.48–0.56)
MDS-primary^1^, n=21	0.68*∗* (0.59–0.75)
E-MDS^2^, n=27	0.56^†^ (0.47–0.65)
L-MDS^3^, n=28	0.54^††^ (0.47–0.62)

^1^MDS-primary: primary myelodysplastic syndrome; ^2^E-MDS: early-stage myelodysplastic syndrome; ^3^L-MDS: late-stage myelodysplastic syndrome; ^4^a.u.: arbitrary units. Note: *∗p*<0.001 rel. to age control; ^†^p<0.05 rel. to MDS-primary; ^††^*p* <0.001 rel. MDS-primary.

## Data Availability

The data used to support the findings of this study are available from the corresponding author upon request.
